# Nucleosome Repositioning: A Novel Mechanism for Nicotine- and Cocaine-Induced Epigenetic Changes

**DOI:** 10.1371/journal.pone.0139103

**Published:** 2015-09-28

**Authors:** Amber N. Brown, Cynthia Vied, Jonathan H. Dennis, Pradeep G. Bhide

**Affiliations:** 1 Center for Brain Repair, Department of Biomedical Sciences, Florida State University College of Medicine, Tallahassee, FL, United States of America; 2 Department of Biological Sciences, Florida State University, Tallahassee, Florida, United States of America; Sanford-Burnham Medical Research Institute, UNITED STATES

## Abstract

Drugs of abuse modify behavior by altering gene expression in the brain. Gene expression can be regulated by changes in DNA methylation as well as by histone modifications, which alter chromatin structure, DNA compaction and DNA accessibility. In order to better understand the molecular mechanisms directing drug-induced changes in chromatin structure, we examined DNA-nucleosome interactions within promoter regions of 858 genes in human neuroblastoma cells (SH-SY5Y) exposed to nicotine or cocaine. Widespread, drug- and time-resolved repositioning of nucleosomes was identified at the transcription start site and promoter region of multiple genes. Nicotine and cocaine produced unique and shared changes in terms of the numbers and types of genes affected, as well as repositioning of nucleosomes at sites which could increase or decrease the probability of gene expression based on DNA accessibility. Half of the drug-induced nucleosome positions approximated a theoretical model of nucleosome occupancy based on physical and chemical characteristics of the DNA sequence, whereas the basal or drug naïve positions were generally DNA sequence independent. Thus we suggest that nucleosome repositioning represents an initial dynamic genome-wide alteration of the transcriptional landscape preceding more selective downstream transcriptional reprogramming, which ultimately characterizes the cell- and tissue-specific responses to drugs of abuse.

## Introduction

Drugs of abuse cause dramatic changes in the brain, often leading to risky and compulsive drug-seeking behavior characterized as addiction. Underlying these behavioral alterations are brain-region and cell-type specific changes in gene expression, mediated in part by epigenetic modifications to DNA and/or histone proteins, as well as local changes in chromatin compaction. Changes in DNA methylation and histone modifications have been reported in brain regions associated with the reward circuitry following exposure to drugs of abuse, such as cocaine, morphine [1, 2], nicotine, amphetamine [3] and cannabis [4]. However, a significant gap exists in our understanding of the mechanisms that license such drug-induced changes in chromatin structure and transcription. Nucleosome repositioning may be one such mechanism [5], although whether it plays a role in modifying DNA accessibility following exposure to drugs of abuse has not been addressed until now.

The nucleosome consists of ~147 base-pairs of negatively-charged DNA wrapped 1.65 times around the positively-charged histone proteins [6], thus enabling the compaction of ~10 meters of linear DNA into ~400μm^3^ nuclei. Compaction of DNA around nucleosomes not only serves to efficiently package the DNA, but also plays a role in permitting or restricting access to DNA by molecules that can regulate gene expression, including transcription factors (TFs). Functionally, a strong DNA-nucleosome interaction makes the nucleosome-bound DNA less accessible to TFs [7, 8] and can serve as a physical barrier to RNA polymerase II progression [9, 10]. In fact, tight nucleosome assemblies are associated with transcriptionally repressed genes and heterochromatic/silent regions of the genome. Conversely, a weak DNA-nucleosome interaction—or nucleosome-depleted region (NDR)—represents loosely packaged or unwrapped DNA, which could permit direct access to the DNA [11]. Thus, whether or not a particular DNA sequence is tightly bound to a nucleosome could affect the probability of successful transcription. Despite the advances in our understanding of DNA-nucleosome interactions and their influence over gene transcription, the role of nucleosome positioning in drug-mediated chromatin and transcriptional changes has not been examined. In this study, we address this question using human neuroblastoma cells (SH-SY5Y) exposed to nicotine or cocaine as a model system.

We report dynamic, widespread, time-resolved and drug-specific changes in nucleosome position at the transcription start site (TSS) and transcription factor binding sites (TFBS) of multiple genes in SH-SY5Y cells following nicotine or cocaine exposure. From these data, we propose that the location along the DNA sequence (e.g. at or away from the TSS or TFBS) where the nucleosome repositioning occurs and duration of the repositioning play important roles in determining drug-induced changes in the chromatin landscape potentiating widespread changes in gene expression.

## Materials and Methods

### Cell culture and drug treatment

We used undifferentiated SH-SY5Y cells (human neuroblastoma cell line; obtained directly from ATCC, Manassas, VA), maintained in culture according to protocols described by ATCC (1:1 F12:EMEM, 10% FBS, 50ug/mL gentamicin). Cells were grown at 37°C until a 150-mm dish was 90% confluent (~2x10^7^cells), at which time the medium was replaced with fresh medium containing nicotine or cocaine (each drug 10μM; Sigma, St. Louis, MO). The cultures were incubated for an additional 10, 60, and 90 min following addition of nicotine; or 5, 20, 40 and 60 min following addition of cocaine, (based on previous reports showing changes in gene and protein expression, described in Technical Considerations below), and fixed with 1% PBS-buffered formaldehyde. Cells treated with an identical volume of the media without nicotine or cocaine were used as controls. Biological replicates were two independently grown cultures treated on nonconsecutive days.

### Cell harvest and DNA purification

At the designated time points, cells were fixed and crosslinked for 10 min in 1% PBS-buffered formaldehyde. The reaction was stopped by incubation for 5 min with 125mM glycine. Nuclei were isolated (0.3M sucrose, 2mM MgOAc_2_, 1% Nonidet P-40, 10mM HEPES (pH7.8)) by centrifugation at 1000 X g for 5 min at 4°C. Bare, unbound genomic DNA and mononucleosomally-protected DNA were isolated from each sample and purified as described previously [12]. A titration of micrococcal nuclease (MNase; 1–1.25U/mL; Worthington Biochemical Corp.) was used to digest bare, unbound DNA and mononucleosomally-protected DNA [12].

### Microarray design and processing

We used NimbleGen’s 12-plex and HD2 design for our custom-designed DNA microarrays that covers 2 kilobase (kb) pair regions flanking the TSS of 858 human genes (S1 Table; GEO accession: GSE71795). Only unique probes were printed on the microarray, and repeats were masked around the TSS. Both forward and reverse DNA strands were printed. The 60-mer oligonucleotide probes were tiled with an average 47 base pair (bp) overlap and each locus on the microarray contains 180 probes with a median spacing of 12.5 bp, allowing us to construct a finely-tuned, high-resolution map of nucleosome positions. 1 microgram of DNA was fluorescently labeled (nucleosomally-protected DNA—Cy3; untreated, bare genomic DNA—Cy5) and hybridized to the custom tiling microarray according to the Nimblegen protocol (Roche).

### Data processing and analysis

Statistical computing and graphical output were performed in the R environment using drawGff.R (publically available at https://github.com/fincher/DrawGff), developed in our laboratory [12, 13]. The microarray data were normalized, the log2 ratio of nucleosomally-protected DNA to untreated, bare genomic DNA signals was computed for each probe, and replicate probes at each locus were averaged. Consistency between 2 independent biological replicates was very high and it is shown as scatterplots (S1 Fig) with corresponding r-values given. To calculate changes in nucleosome occupancy on a per-locus basis, all probe intensities across a 2kb TSS for each condition were compared to the probe intensities across the same region during control conditions and a correlation value was calculated (r<0.7) for each of the 858 genes represented on the array. Each biological replicate was compared to each control condition and only those loci with an r<0.7, and that were common among all comparisons, were reported as significantly changed. We have previously demonstrated the significance of the 0.7 threshold using a wavelet-based ANOVA (WANOVA) method, which is capable of detecting changes of very small magnitudes in comparing functional responses [12]. Computational model scores for nucleosome occupancy were derived from algorithms previously described [14]. Per-gene correlations to the model were calculated for each condition compared to control conditions, and the difference between these correlations and the model were calculated to give difference scores for each of the 858 genes on the array. Analyses of gene ontology were performed using the DAVID Gene Classification tool [15, 16], using the molecular function category with Bonferroni correction for multiple comparison correction.

## Results

### Technical Considerations

Undifferentiated SH-SY5Y cells used in our studies express α and β subunits of nicotinic acetylcholine receptors, D1- and D2-like dopamine (DA) receptors, the dopamine transporter (DAT), tyrosine hydroxylase (TH)[17], and exhibit ERK1/2 signaling following nicotine treatment [18]. In culture conditions, these cells take-up DA via DAT—a process that can be pharmacologically blocked by cocaine [19–21]. Thus, undifferentiated SH-SY5Y cells express signaling molecules mediating responses to nicotine and cocaine and therefore are suitable *in vitro* models to study the effects of these drugs on nucleosome repositioning. The concentrations of cocaine and nicotine, and the durations of exposure to each drug used in the present study are chosen to be consistent with parameters known to induce changes in Ca2+ influx, receptor activation and gene expression in multiple cell lines as well as animal models, with low cytotoxicity [8, 22–24]. Therefore, we believe that the parameters used here offer significant advantage for extrapolation of the findings to multiple cell types and the intact organism. We recognize that the undifferentiated SH-SY5Y cells do not fully represent the complexities of heterogeneous neuronal populations in the intact brain because mature neurons can exhibit shorter linker DNA compared to other cell types [25, 26]. However, this limitation notwithstanding, at the present time the SH-SY5Y cells used here appear to be excellent model systems to study the effects of nicotine or cocaine on undifferentiated, proliferating cells such as the precursor cells that predominate the fetal brain [27–31]. Therefore, using the undifferentiated SH-SY5Y cells serves a valuable purpose—it offers insights into chromatin remodeling mechanisms that could be associated with changes in the brain and behavior produced by fetal drug exposure.

Our microarrays were designed for the specific purpose of investigating changes in nucleosome positioning [12]. These arrays cover a 2 kb stretch centered on the TSS of 858 human genes integrally related to general cellular and metabolic processes, and at least 53% of which are expressed in the brain (GeneAnalytics [32]). This dataset includes genes for transcription factors, (eg. JUN, FOS, EP300, CREBBP), neurotrophic factors, (eg. CNTFR, BDNF), cell adhesion molecules, (eg. NCAM1, ICAM1, MCAM) and neurotransmitter receptors (eg. GABBR1, HTR2B), in addition to a variety of growth factors, signaling molecules and proteins involved in cell division, inflammation and immune responses (listed in S1 Table). The 2 kb region centered on the TSS offers additional advantages by focusing on promoter regions, which most directly regulate transcriptional activity. The unique characteristics of the SH-SY5Y cells and the microarray discussed above have permitted the very first description of the effects of drugs of abuse on chromatin structure at the level of DNA-nucleosome interactions.

### Nicotine and nucleosome repositioning

**Fig 1:**
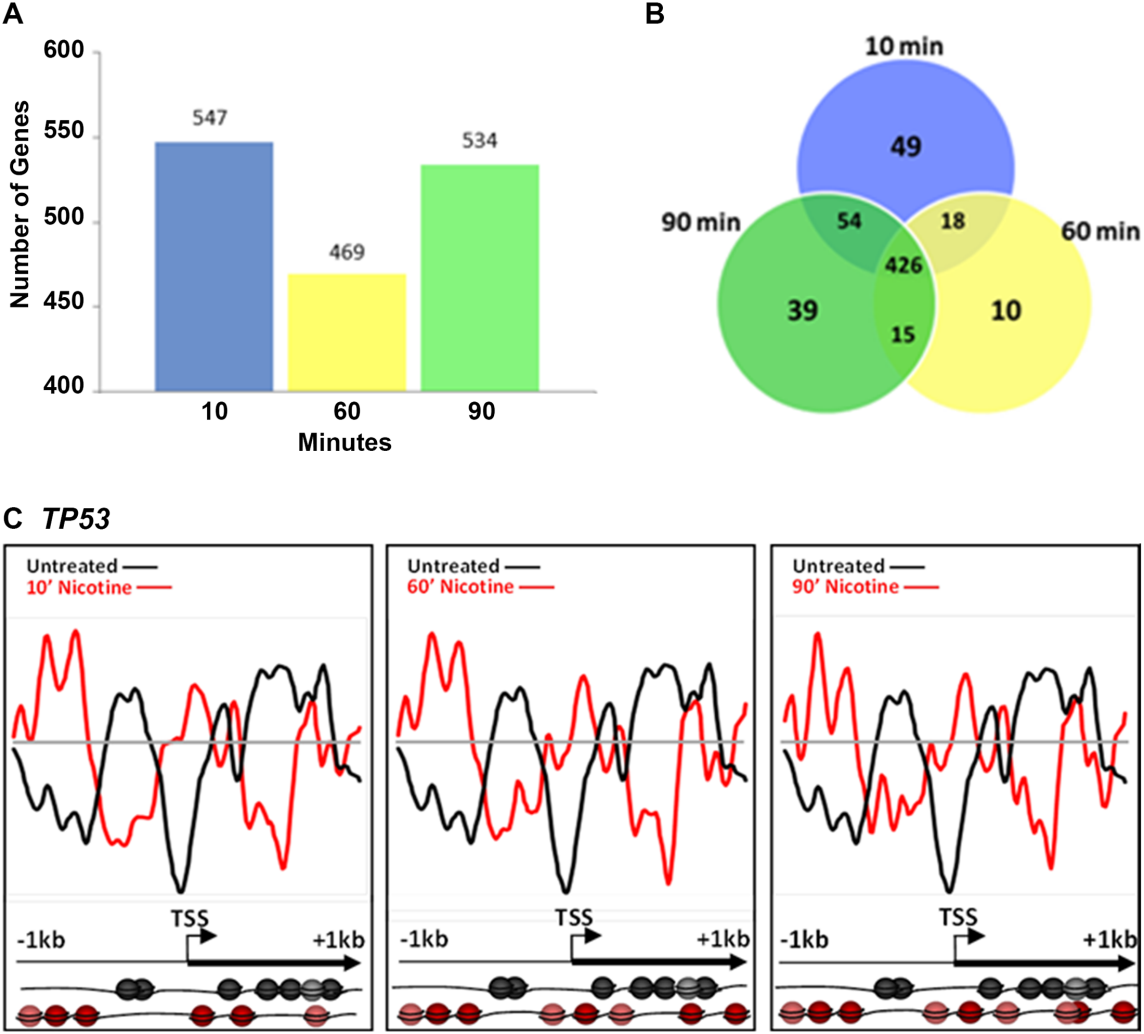
Nicotine-induced nucleosome repositioning. Comparative genome hybridization of mononucleosomally-protected DNA reveals a large-scale repositioning of nucleosomes following nicotine treatment. (A) Number of loci with significant nucleosome repositioning at 10, 60 and 90 minutes. (B) Number of genes showing nucleosome repositioning that was unique to each time point and common to multiple time points is shown. Common changes were in the majority with 426 out of 858 loci showing changes at every one of the time points analyzed. Some changes are unique to early or late time points (49 loci specific for 10 min, 10 loci for 60 min, and 39 loci for 90 min). (C) Nucleosomes are repositioned upstream of the transcription start site (TSS) and throughout the 5’ end of the *TP53* gene following 10, 60 and 90 min of nicotine exposure. Changes in nucleosome occupancy at the *TP53* promoter were observed at 10 min and persisted through the 60 and 90 min time points with only minor modifications. The x-axis represents the genomic position showing 2 kb centered on a TSS. The y-axis is the log_2_ ratio of mononucleosomally-protected DNA to genomic DNA signal at each probe on the microarray. For all figures, changes in nucleosome position can be appreciated by comparing the divergence of the red and black lines from the grey line at the center of each image. The grey line represents theoretical zero likelihood of nucleosome occupancy. The black line is the basal nucleosome occupancy signal in the drug naïve state. The red line is the nucleosome occupancy signal following drug exposure. In each image, deflections of the black and red lines above the grey line indicate nucleosome occupancy, and deflections below the grey line indicate nucleosome depletion. Nucleosome positions relative to the TSS and coding sequence of the gene in the drug naïve (black) and drug exposed (red) states are further illustrated pictorially at the bottom of the figure. Each sphere (black or red) represents a nucleosome.

Following treatment of SH-SY5Y cells with 10μM nicotine, significant genome-wide changes in nucleosome positions were observed for each time point examined (10, 60 and 90 min), relative to the untreated controls (basal or drug naïve positions). Across all time points examined, 611 genes (71%) developed significant nucleosome distribution changes, showing that these changes are widespread. As early as 10 min following nicotine treatment, nucleosomes in the -1000 to +1000 bp region of 547 genes (64%) were significantly repositioned (r<0.7; Fig 1A). By 90 min, nucleosomes were restored to their basal state at 77 genes, whereas 534 genes (63%) still had nucleosome organization with significant repositioning compared to the basal state (Fig 1B). Thus, nicotine-induced nucleosome repositioning was rapid and long lasting. Interestingly, the frequency and positions of these changes are similar to nucleosome repositioning events described by us in a different cell line following virus reactivation [12].

We performed a detailed analysis of nicotine-induced changes in nucleosome position in the *TP53* (tumor suppressor protein P53) gene at upstream regions of the TSS and throughout the 5’ region at 10, 60 and 90 min durations (Fig 1C). Following 10 min nicotine treatment, nucleosomes were repositioned across the 2kb examined region (r = -0.4233 +/- 0.9964), including the promoter region and the TSS. These changes persisted throughout the 60 min (r = -0.2211 +/- 0.1478) and 90 min (r = -0.1205 +/- 0.1739) time point (Fig 1C, nucleosome models). Representative examples of nicotine-induced changes in nucleosome repositioning lasting until the 90 min time point are also shown for *PDGFA*, *HDAC10* and *LMNB1* (S2 Fig). We chose to illustrate changes in the *TP53* gene in detail because 10uM nicotine has been shown to increase expression of p53 after 24 hours in human oral keratinocytes [33]. Additionally, TP53 is involved in the metabolism of dopamine [34], a neurotransmitter intimately associated with reward and addiction pathways and the expression of *TP53* is elevated in various brain regions in an animal model of adolescent nicotine exposure [35].

### Cocaine and nucleosome repositioning

**Fig 2:**
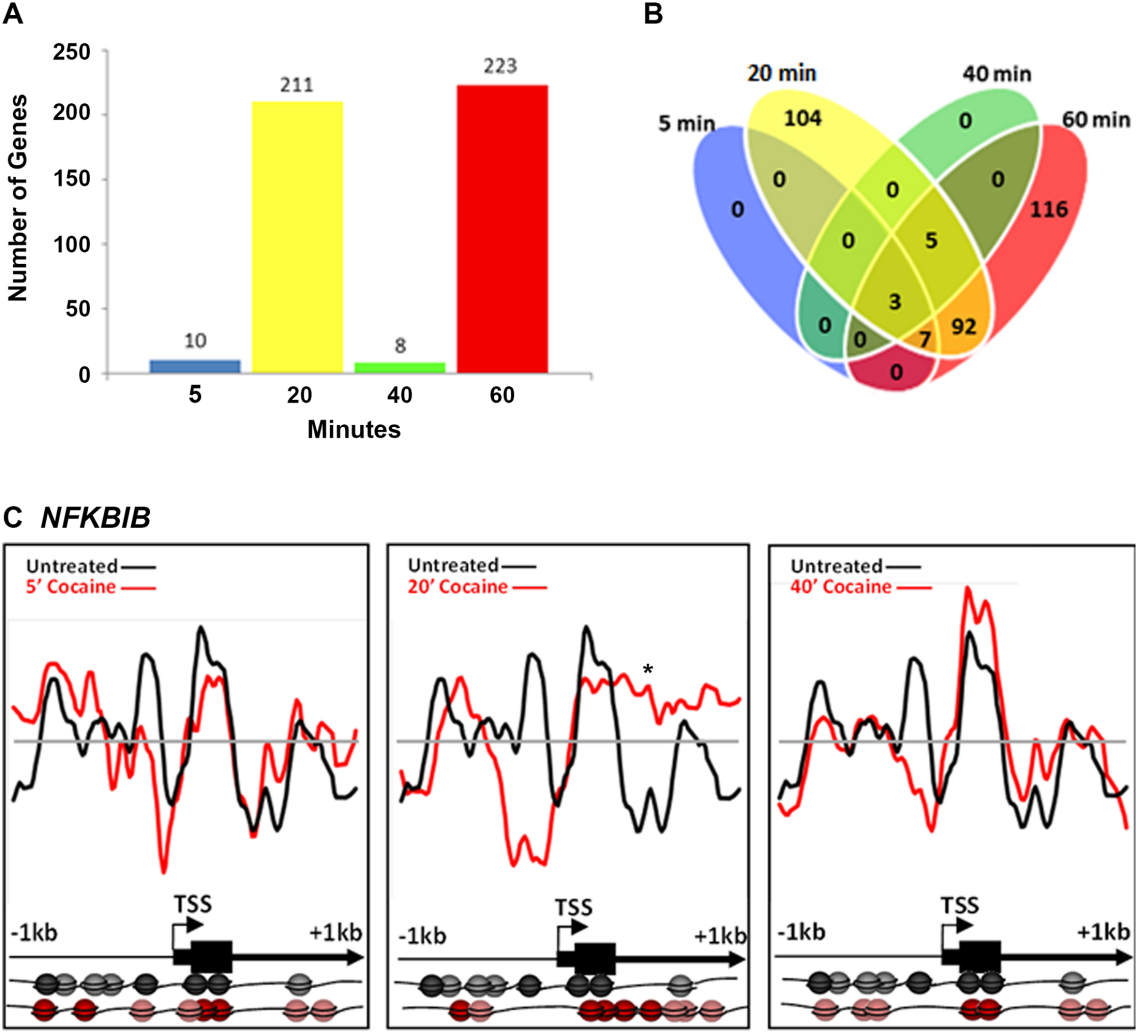
Cocaine-induced nucleosome repositioning. Comparative genome hybridization of mononucleosomally-protected DNA reveals a large-scale repositioning of nucleosomes following cocaine treatment. (A) The number of loci with significant changes in nucleosome positions fluctuates with the duration of exposure. Between 5 and 20 min, there is a significant increase in the number of loci with nucleosome repositioning (from 10 at 5 min to 211 at 20 min). However, by 40 min, the majority of the nucleosomes return to their basal positions such that only 8 loci continue to show nucleosome repositioning. At 60 min, 223 loci show significant repositioning of nucleosomes. (B) Number of genes showing nucleosome repositioning that was unique to each time point and common to multiple time points is shown. The changes detected at 5 and 40 min were common across the 20 and 60 minute time points. Some changes are unique to early or late time points (104 loci specific for 10 min, 116 loci for 60 min). (C) Nucleosome repositioning upstream of the transcription start site (TSS) and throughout the 5’ end of the *NFKBIB* gene following 5, 20 and 40 min of cocaine exposure. Nucleosome positions relative to the TSS and coding sequence of the *NFKBIB* gene in the drug naïve (black) and cocaine exposed (red) states are further illustrated pictorially at the bottom of the figure. Each sphere (black or red) represents a nucleosome. Nucleosome positions do not diverge noticeably from the basal positions at 5 min following the cocaine treatment. By 20 min, there is significant repositioning, such that nucleosomes are evicted upstream of the TSS. However, the nucleosomes return to the basal position by the 40 min (60 min identical to 40 min; data not shown). The x-axis represents the genomic position showing 2 kb centered on a TSS. The y-axis is the log_2_ ratio of mononucleosomally-protected DNA to genomic DNA signal at each probe on the microarray.

Cocaine (10μM) also induced changes in nucleosome position, although less robustly than did nicotine. Interestingly, the number of genes showing nucleosome repositioning fluctuated over time. Following 5, 20, 40 and 60 min cocaine exposure, nucleosome positions in 10, 211, 8 and 223 genes or 1%, 25%, 1% and 26% of the total, respectively, were significantly remodeled (r<0.7; Fig 2A). Among the “early” changes (5, 20 and 40 min time points) 48% occurred in genes unique to the 20 min time point and nucleosomes in these genes had returned to their basal state by 40 min. A second-wave of nucleosome repositioning occurred at 60 min, with 116 genes or 52% of the changes being unique to the 60 min time point (Fig 2B).

We analyzed in detail cocaine-induced changes in nucleosome occupancy in the *NFKBIB* gene (IKBB; inhibitor of NFkB translocation to the nucleus) at upstream regions of the TSS and throughout the 5’ region (Fig 2C). We chose to illustrate the changes in this gene because it shows increased H3 acetylation and is upregulated in the nucleus accumbens, in the brain’s reward pathways, following cocaine exposure in rodents [36]. No significant changes in nucleosome position were detected in this gene following 5 min cocaine exposure (r = 0.5025 +/- 0.0543). However, following 20 min exposure (r = -0.0478 +/- 0.0455), nucleosomes were repositioned, (asterisk *; Fig 2C) from the promoter region upstream of the TSS and occupying previously vacant loci downstream of the TSS. By 40 min (r = 0.6053 +/- 0.0400) and 60 min (0.1813 +/- 3149), the majority of the nucleosomes had returned to the basal positions (Fig 2C shows 40 min data only because 60 min was similar to 40 min).

### Comparison between nicotine- and cocaine-specific changes

**Fig 3:**
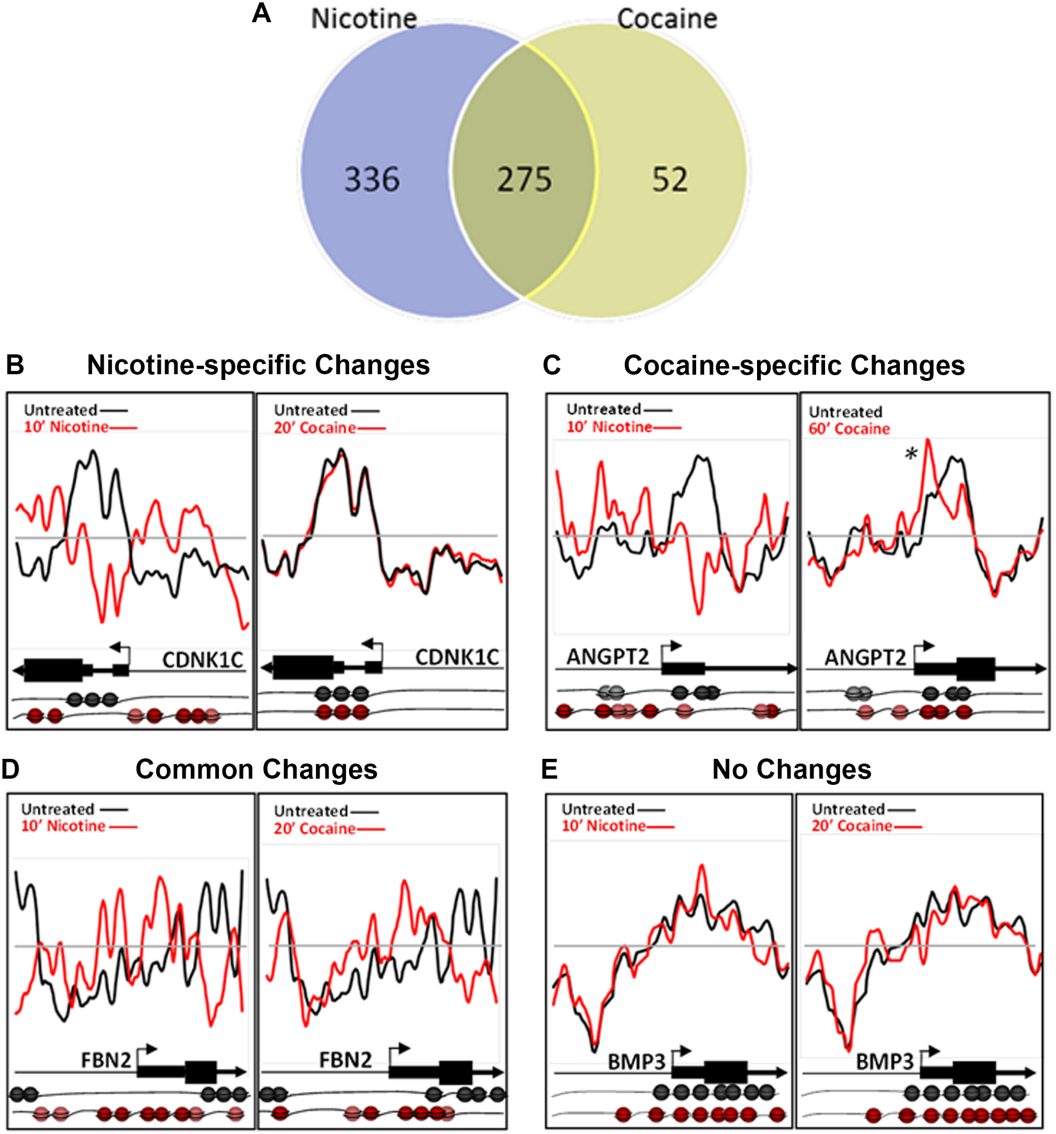
Cocaine- and nicotine-induced nucleosome repositioning comparisons. (A) Changes in nucleosome repositioning that were unique to nicotine or cocaine, and common to both the drugs. (B-E) Nucleosome repositioning in response to nicotine or cocaine exposure is shown. (B) Nucleosome repositioning upstream of TSS, at the TSS and at the beginning of the coding sequence in the *CDNK1C* gene around the TSS was induced by nicotine and not by cocaine (nicotine-specific). Nicotine exposure positioned nucleosomes upstream of the TSS, depleted the nucleosomes at the TSS, and positioned nucleosomes at the start of the coding sequence. On the other hand, cocaine exposure for 20 min, did not produce changes in nucleosome position compared to the drug naïve state. (C) Nicotine and cocaine both induced nucleosome repositioning upstream of TSS, at the TSS and at the beginning of the coding sequence in the *ANGPT2* gene. However, 60 min cocaine-specific changes were detected at the +1 nucleosome (asterisk), just downstream of the *ANGPT2* TSS. The 60 min nicotine-induced nucleosome repositioning at *ANGPT2* is similar to the 20 min time point and therefore is not shown. (D) Changes common to nicotine (10 min) and cocaine (20 min) just upstream and downstream of the TSS of *FNB2* gene. (E) Neither nicotine (10 min) nor cocaine (20 min) produced nucleosome repositioning across the promoter and 5’ region of *BMP3*. Nucleosome positions relative to the TSS and coding sequence of each gene in the drug naïve (black) and drug exposed (red) states are further illustrated pictorially at the bottom of the figure. Each sphere (black or red) represents a nucleosome.

Comparative analysis of nicotine- and cocaine-induced changes reveals nucleosome repositioning events that are unique to each drug and common to both. Overall, approximately half (55%) of the nicotine-induced changes were unique to nicotine, while only 16% of the cocaine-induced changes were unique to cocaine (Fig 3A). Nicotine-specific changes were seen in the *CDKN1C* gene (Cyclin-Dependent Kinase Inhibitor 1C; Fig 3B), which is hypermethylated and downregulated in human bronchial epithelial cells following cigarette condensate exposure [37, 38]. As shown for the 10 min nicotine exposure time point, nucleosomes were repositioned to occupy the promoter region, just upstream of the TSS, representing a transcriptionally-restrictive configuration, relative to the basal condition (Nicotine r = 0.5130 +/- 0.207; Cocaine r = 0.7495 +/- 0.0257). Cocaine-specific changes were detected for *ANGPT2* (Angiopoietin 2; Fig 3C; Nicotine r = 0.0797 +/- 0.0929; Cocaine r = 0.5130 +/- 0.1715). Nucleosomes were enriched and retained at the TSS following cocaine exposure only (Fig 3C, asterisk *), however, the effect of cocaine on expression of *ANGPT2* in any cell type is unknown. We identified genes with nucleosome repositioning events common to nicotine and cocaine, as shown for *FBN2* (Fibrillin 2), which has increased nucleosome occupancy both upstream and downstream of the TSS (Fig 3D; Nicotine r = -0.1343 +/- 0.0717; Cocaine r = 0.1718 +/- 0.0281). We also identified genes with no significant changes in nucleosome position in response to either drug, shown for *BMP3* (Bone morphogenic protein 3), which maintains a transcriptionally restrictive configuration (Fig 3E; Nicotine r = 0.8087 +/- 0.0872; Cocaine r = 0.8465 +/- 0.0084). There are no reports in the literature as to the transcriptional changes elicited in these genes following either drug exposure. Therefore, *CDNK1C*, *ANGPT2* and *FBN2* are potential novel genes that are regulated by cocaine or nicotine exposure.

Analysis of gene ontology reveals that nicotine-specific nucleosome remodeling events occur within the promoter and 5’ region of genes enriched for multiple distinct molecular functions (Table 1). Among the loci with unique changes following nicotine treatment, enrichment was observed in genes associated with acetyltransferase and cyclin-dependent kinase activities. Cocaine-specific changes were not significantly enriched for any specific molecular function.

**Table 1:** Gene Ontology Enrichment.

Nicotine only	Fold enrich.	Adj. p-value
GO:0004861:CDK inhibitor activity	16.26	3.02E-05
GO:0004407:histone deacetylase activity	12.39	3.27E-05
GO:0033558:protein deacetylase activity	12.39	3.27E-05
GO:0016538:CDK regulator activity	10.93	8.36E-05
GO:0005524:ATP binding	1.57	8.34E-05
GO:0019213:deacetylase activity	10.32	1.24E-04
GO:0032559:adenyl ribonucleotide binding	1.55	1.38E-04
GO:0004713:protein tyrosine kinase activity	3.08	1.80E-04
GO:0001883:purine nucleoside binding	1.49	4.10E-04

Nicotine-specific changes in nucleosome position are represented in molecular function gene ontology (GO) terms, ranked in descending order of magnitude. Cocaine-specific changes did not reach statistical significance for any GO category. All time points for nicotine or cocaine treatment used in our study were considered together. Fold enrich., fold enrichment. Adj. p-value, Bonferonni corrected p-value.

### DNA sequence is a *cis*-factor in drug-induced nucleosome redistribution

**Fig 4:**
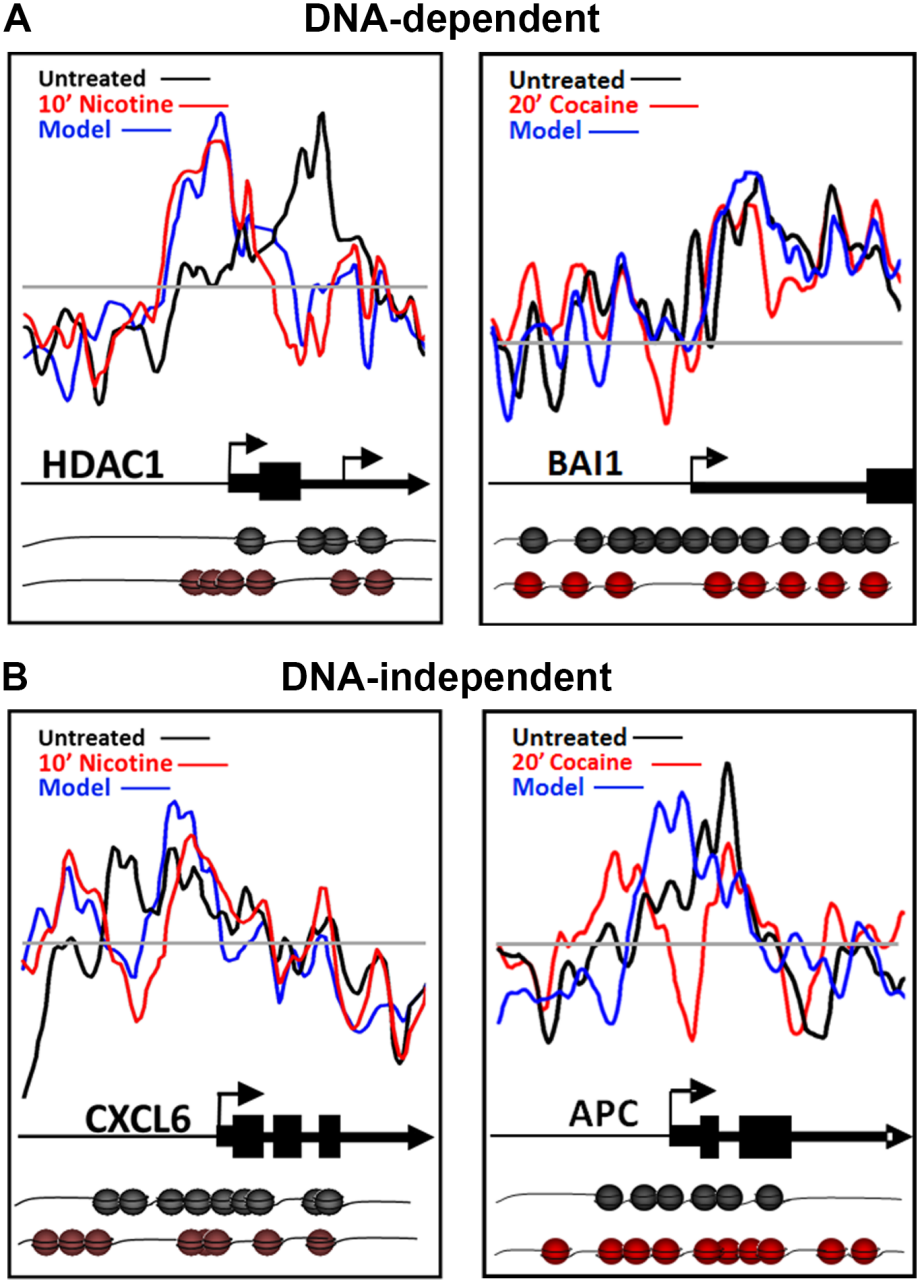
Role of DNA sequence in nicotine- and cocaine-induced nucleosome repositioning. (A) DNA-directed nucleosome repositioning events following nicotine or cocaine treatment are shown for the *HDAC1* and *BAI1* genes. For both examples, nucleosomes were repositioned upstream and downstream of the TSS, according to the DNA-directed model. (B) DNA-independent nucleosome repositioning events following nicotine or cocaine treatment are shown for the *CXCL6* and *APC* genes, with significant remodeling events in the upstream regions and TSS of both genes. Nucleosome positions predicted by the DNA-directed model are shown as a blue line. Nucleosome illustrations (black—basal; red—nicotine treated) depict the changes in individual nucleosome positions, relative to the TSS and coding sequence of the gene model above.

Nucleosome occupancy can often be predicted *in silico* based on the DNA sequence underlying the DNA-nucleosome interactions, the so called DNA-directed nucleosome occupancy model [14, 39]. We compared nucleosome occupancy scores between basal and drug-treated conditions to the DNA-directed nucleosome occupancy model. Following nicotine or cocaine treatment, approximately half (50%) of the nucleosome redistributions were consistent with the DNA-directed nucleosome occupancy model, regardless of the time point examined. Representative plots of nucleosome repositioning following nicotine or cocaine treatment, and their correlation to the DNA-directed nucleosome occupancy model are shown in Fig 4A for *HDAC1* (histone deacetylase 1; Nicotine versus controls r = 0.1787 +/- 0.9965; Nicotine versus model r = 0.8210 +/- 0.0382) and *BAI1* (Brain angiogenesis-inhibitor 1; Cocaine versus controls r = 0.4512 +/- 0.0821; Cocaine versus model r = 0.5625 +/- 0.0007), respectively. Examples of nucleosome repositioning events that do not fit the DNA-directed nucleosome occupancy model, and therefore are DNA-independent, are shown in Fig 4B for *CXCL6* (chemokine C-X-C motif, ligand 6); Nicotine versus controls r = 0.4837; Nicotine versus model r = 0.6190 +/- 0.0085) and *APC* (anaphase promoting complex; Cocaine versus controls r = 0.0550; Cocaine versus model r = 0.3488 +/- 0.0280). These results suggest that the occurrence of DNA-directed and DNA-independent changes in nucleosome positioning are gene-specific, suggesting a role for the underlying DNA sequence as a *cis*-factor in directing half of the nucleosome repositioning events detected in response to nicotine and cocaine exposure.

### Nucleosome repositioning alters the regulatory landscape of the *EGR1* promoter following nicotine exposure

**Fig 5:**
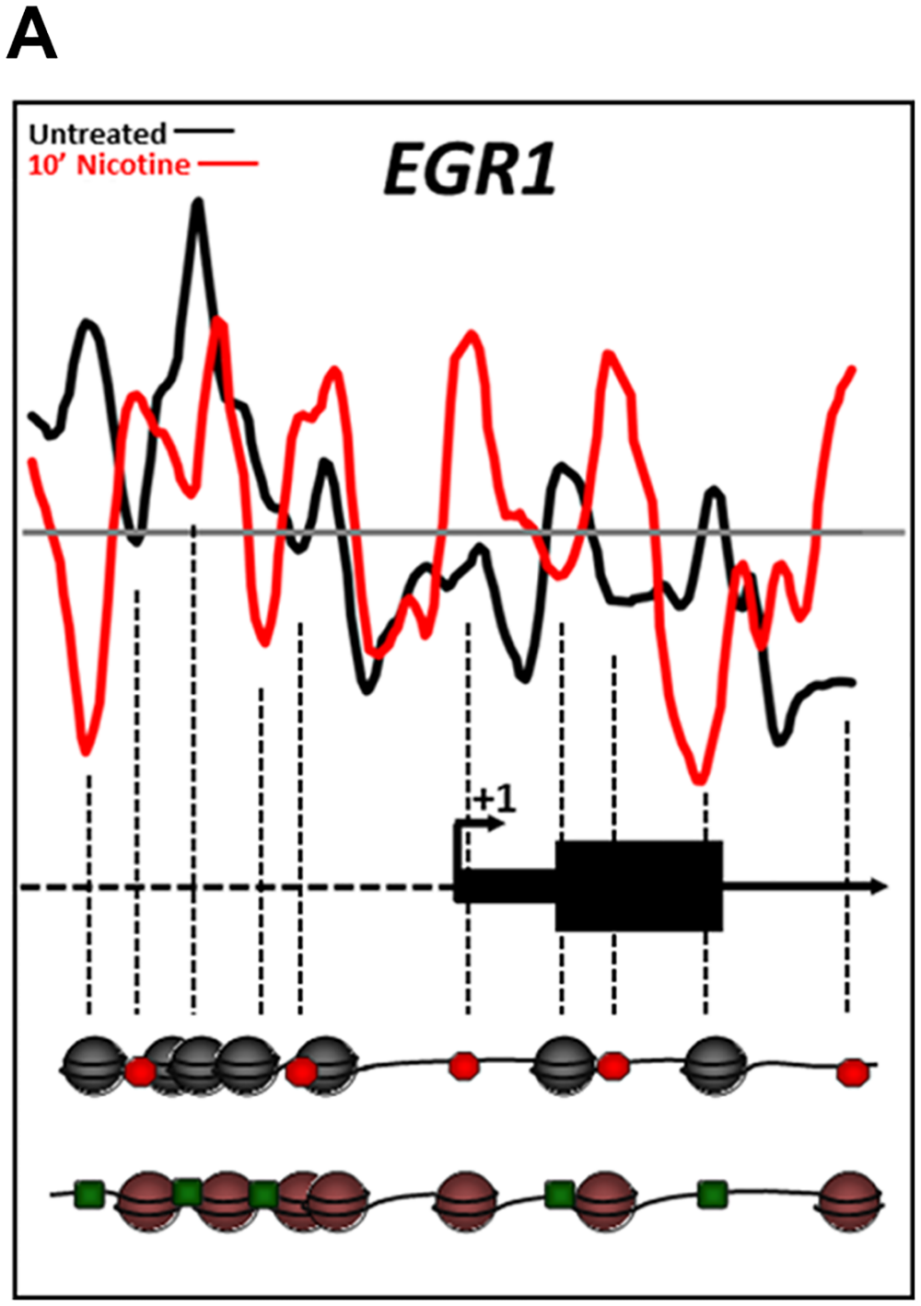
Changes in nucleosome position at the *EGR1* promoter region induced by nicotine exposure correlate with induction of the *EGR1* gene. Nucleosome positions relative to the transcription start site in the drug naïve (black) and nicotine exposed (red) states are further illustrated pictorially at the bottom of the figure. Each sphere (black or red) represents a nucleosome. The dashed vertical lines indicate loci of changes in nucleosome occupancy from the plot down to the nucleosome model. Based on the data from the ENCODE Consortium the red octagons indicate binding sites for transcription factors (TFs) known to repress *EGR1* expression whereas the green octagons represent binding sites for permissive TFs. Nucleosome positioning in the drug naïve basal state favored binding of repressive transcription factors (red octagons), such as LYF1/IKAROS, CTCF and EGR1 itself). However, nicotine exposure repositioned the nucleosomes such that binding sites for transcription factors such as FOS, SRF, JUNB, ZNF263, RELA, E2F1, TR4 (green squares) became depleted of nucleosomes consistent with a nucleosome configuration permissive for *EGR1* induction.

Nucleosomes are typically depleted just upstream of and at the TSS of promoters, as well as other *cis*-regulatory elements such as TFBS of “active” genes [40, 41]. Genetic loci with significant nucleosome repositioning within these regulatory regions are believed to be remodeled in preparation for a transcriptional response, repositioning nucleosomes either to inhibit or permit the binding of cell-specific *trans-*acting factors. As ~50% of the nicotine- and cocaine-induced nucleosome positioning changes we identified were classified as DNA-directed, we examined the changes at the TSS and TFBS in genes known to be regulated in the central nervous system following nicotine or cocaine exposure. One such gene is the immediate-early gene (IEG) *EGR1* (early growth responsive-1; *ZIF268*). *EGR1* is a transcription factor [42, 43] that is rapidly upregulated in the brain or in a neuronal cell line within minutes of nicotine or cocaine exposure [44, 45]. In fact, nicotine was shown to cause upregulation of *EGR1* expression in the same cell line, SH-SY5Y, used in our study (Dunckley and Lukas, 2003). Induction of *EGR1* by several TFs has been well characterized [46]. Using transcription factor ChIP data for the *EGR1* locus, which was thoroughly mapped by the ENCODE Project Consortium [47], we demonstrate that nicotine treatment results in repositioning of nucleosomes in a manner consistent with rapid induction of *EGR1* expression (Fig 5). Nucleosomes were depleted just downstream of the TSS and robustly positioned at the +1 nucleosome position following 10 min nicotine treatment (Fig 5), features characteristic of transcriptionally-active genes [11]. In addition, nucleosomes were depleted from TFBS known to induce gene expression (binding sites for FOS, SRF, JUNB, ZNF263, RELA, E2F1, TR4; green squares in Fig 5) and/or relocated to occupy binding sites for more repressive TFs (LYF1/IKAROS, CTCF and EGR1; red octagons in Fig 5). Cocaine exposure, however, did not result in significant remodeling of the *EGR1* locus. This may reflect the fact that this cell culture model does not sufficiently recapitulate the dopamine transporter blockade caused by cocaine *in vivo*, which may also explain the finding of no significant enrichment of genes with specific molecular functions following cocaine treatment (Table 1). Nonetheless, these data support the current concepts of transcriptional induction of *EGR1* following nicotine treatment, specifically in the SH-SY5Y cell line, and point to nucleosome repositioning as a mechanism for licensing the rapid transcriptional response.

**Fig 6:**
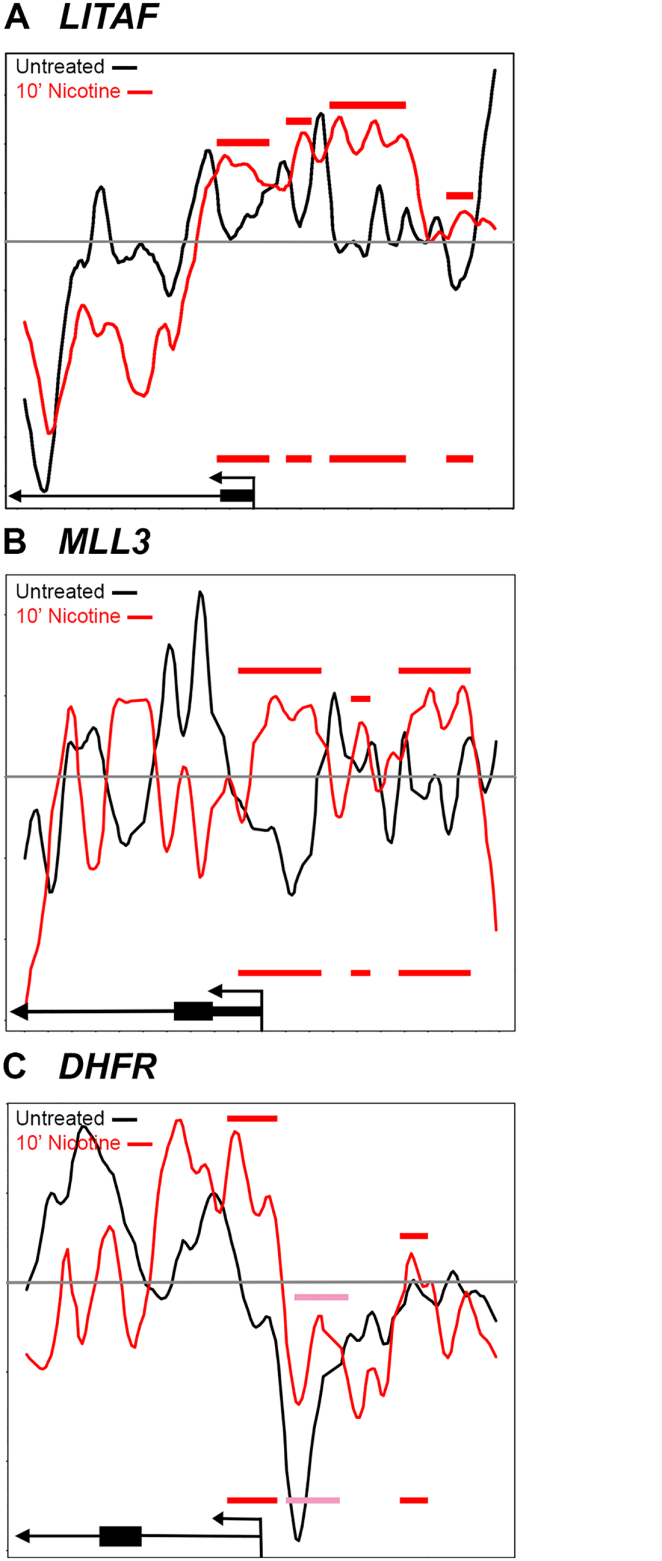
Nicotine-induced changes in nucleosome occupancy at the *LITAF*, *MLL3* and *DHFR* promoter regions. (A) Nucleosome positioning at the *LITAF* and (B) *MLL3* promoter after 10 minute nicotine exposure. Increased nucleosome occupancy in the nicotine treated cells (red line) relative to control cells (black line) is indicated by red bars. Nucleosome occupancy is enhanced, relative to control, at the regions just upstream of the TSS in both *LITAF* and *MLL3*, consistent with repressed transcription. (C) Nucleosome occupancy at *DHFR* is also enhanced at the TSS, above baseline and is enhanced upstream of the TSS, relative to the control, but not above baseline (pink bar). The 60 and 90 minute time points were similar to the 10 minute nicotine time point and therefore, were not shown. Nucleosome positions relative to the transcription start site in the drug naïve (black) and nicotine exposed (red) states are further illustrated pictorially at the bottom of the figure. Red and pink bars represent increased nicotine-induced nucleosome occupancy upstream of the TSS and are replicated on the pictoral illustrations at the bottom of the figure.

In addition to *EGR1*, we examined nucleosome positioning for three other genes that were found to have altered gene expression in the SH-SY5Y cells following nicotine exposure. In contrast to *EGR1*, *DHFR* (Dihydrofolate reductase), *LITAF* (Lipopolysaccharide-induced TNF-alpha factor) and *MLL3* (*KMT2C*; Lysine-specific methyltransferase 2C) were all transcriptionally repressed in response to nicotine [44]. Nucleosome occupancy is enhanced, relative to control, at the regions just upstream of the TSS in both *LITAF* and *MLL3*, consistent with repressed transcription (Fig 6A and 6B). Nucleosome occupancy at *DHFR* is also enhanced at the TSS, above baseline (Fig 6, grey line), and is enhanced upstream of the TSS, relative to the control, but not above baseline (Fig 6C, pink bar). Since transcription of *DHFR* is repressed in response to nicotine, the change of nucleosome occupancy, relative to control, might be more critical than the baseline. Interestingly, in the study by others [44], SH-SY5Y cells were treated with 1mM of nicotine for one hour, while we used 10μM for all time points. The increased concentration of nicotine could result in a more dramatic repositioning of the nucleosomes for *DHFR* and the other genes. Despite this difference in procedure, we see changes in nucleosome positioning in *EGR1*, *LITAF*, *MLL3* and possibly *DHFR*, beginning at the 10 minute time point that are consistent with transcriptional changes following one hour nicotine exposure in the SH-SY5Y neuroblastoma cell line.

## Discussion

We report a novel mechanism for nicotine and cocaine induced regulation of chromatin structure at the level of DNA-nucleosome interactions that involves nucleosome repositioning along specific DNA sequences in SH-SY5Y cells. Both drugs rapidly and globally remodel nucleosome positions along DNA, notably within regulatory regions of promoters and at the TSS of genes. Nucleosome repositioning was both drug-specific and time-resolved, with changes throughout more than half of the loci examined, some nucleosome repositionings were unique to each drug while others were shared by both of the drugs. In addition, the intrinsic DNA sequence played a significant role in determining nucleosome positioning, with approximately 50% of all genes showing changes that were highly correlated with the DNA-directed model. The contribution of DNA sequence to nucleosome position may have implications for gene expression because nucleosome occupancy at conserved noncoding sequences and TFBS is functionally correlated to transcriptional activity in yeast [5], plant [48] and human cells [49].

The DNA-independent nucleosome repositioning events likely occur in a class of genes in which ATP-dependent chromatin remodeling complexes are required to direct changes in nucleosome position. The SWI/SNF chromatin remodeling complex MSK1 (also containing Brg1) is recruited to and phosphorylates histone H3 on specific nucleosomes within induced IEG promoters [50], thus reinforcing the importance of nucleosome position in evoked transcriptional responses. Recent studies have shown that expression of neuronal-specific subunits of the ATP-dependent SWI/SNF nucleosome remodeling complex BAF (Brg1-associated factor complex) is required for dendritic outgrowth, synaptic plasticity and various aspects of neuronal function and development [51–53]. As such, these chromatin remodeling complexes represent likely candidates underlying nucleosome dynamics and neuronal plasticity induced by drugs of abuse.

Overall, nicotine led to more widespread, robust and sustained changes in nucleosome position relative to cocaine. These longer-lasting changes may allow for protracted or poised transcriptional activity and/or additional epigenetic modifications to the exposed DNA and/or histone proteins. Poised or paused RNA polymerase II has been identified as a factor involved in nicotine’s “priming” effect on subsequent exposure to other drugs of abuse, known as the nicotine gateway hypothesis [54, 55]. Given that the +1 nucleosome position has been shown to influence poised polymerase and related transcriptional activity [9], our data provoke intriguing possibilities as to the drug-specific nature and influence of nucleosome repositioning on gene expression.

Cocaine exposure resulted in far fewer and more transient changes in nucleosome remodeling, as evidenced by the rapid return of many nucleosomes to their basal or drug naïve positions within 40 min of exposure. Intriguingly, we detected a second, distinct nucleosome remodeling event at 116 loci following 60 min cocaine exposure. It is possible that differences observed between the 20 min and 60 min cocaine exposures represent two different waves of chromatin remodeling—the first licensing ERK-mediated IEG induction, and the second IEG-mediated delayed-response gene induction. This type of immediate and delayed transcriptional response is common to neuronal signaling related to learning, memory, drug tolerance and sensitization [56] and such events are believed to mediate the changes in neuronal plasticity and behavior following cocaine exposure [57]. Thus, the two waves of nucleosome repositioning events observed following cocaine treatment may represent these types of temporal changes in transcriptional activity at immediate early- versus delayed-response genes. Alternatively, prolonged exposure to cocaine (60 min) may indeed initiate a second, distinct nucleosomal response, similar to the gene expression and chromatin modifications reported to be unique to chronic drug exposure *in vivo* [4]. Lastly, it is possible that this cell culture model, undifferentiated SH-SY5Y cells, may not be ideal for correlating *in vivo* changes, as it does not provide the same synaptic environment for cocaine’s blockade of the dopamine transporter.

Not all of the nucleosome repositioning events promoted a transcriptionally favorable configuration, such as that shown for *EGR1* in Fig 5 and *NFKBIB* in Fig 2C. For example, *TP53*, a well-characterized tumor-suppressor protein, is downregulated following nicotine exposure [58]. Consistent with this finding, nicotine induced a nucleosome organization consistent with transcriptional suppression in *TP53*, with well-positioned nucleosomes at the TSS and multiple TFBS. Nicotine’s effects on *TP53* may also have a direct consequence on reward pathways, as recent evidence indicates that inactivation of *TP53* leads to an induction of tyrosine hydroxylase biosynthesis in dopaminergic neurons [34]. Similarly, nicotine caused transcriptionally restrictive changes in nucleosome positions at the *CDNK1C* (Fig 3B), *LITAF and the MLL3* promoters (Fig 6), by repositioning nucleosomes to the region upstream of the TSS. These changes are consistent with downregulation of gene expression of *CDNK1C*, *LITAF* and *MLL3* following nicotine exposure [37, 44].

We provide here the first evidence of nucleosome positioning as an early pre-transcriptional event following exposure to nicotine or cocaine at or near TSS and TFBS of multiple genes. We propose that these events likely represent early genomic responses, which expose or protect DNA to the effects of cell-type specific *trans*-acting factors that lead to behaviorally relevant cell- and tissue-specific changes in the chromatin landscape. Sequencing-based technologies, which require less input material, will allow for the investigation of these changes in terminally differentiated cell populations *in vivo*. Understanding the long-term consequences of nucleosome repositioning and the mechanisms by which drugs of abuse can remodel chromatin at this level may provide insight into the lasting neurological, biochemical and often transgenerational effects of drugs of abuse [28, 31, 59–61].

## Supporting Information

S1 Fig Biological Replicate Consistency. (A–H) Scatterplots for biological replicates for each drug and every time point demonstrate the correlation between replicates shown by the corresponding r-values. The x- and y-axis represent the log2 ratio of nucleosomally-protected DNA to genomic DNA signal at each probe on the microarray.
(TIF)

S2 Fig Nicotine-induced Nucleosome Repositioning. Persistent repositioning of nucleosomes is seen for (A) *PDGFA*, (B) *HDAC10* and (C) *LMNB1* genes following 10, 60 and 90 min of nicotine exposure. Changes in nucleosome position are depicted as the divergence of the red and black lines. The black line is the basal nucleosome occupancy signal in the drug naïve state. The red line is the nucleosome occupancy signal following nicotine exposure. Changes in nucleosome occupancy for all three genes were observed at 10 min and persisted through the 60 and 90 min time points with few modifications.
(TIF)

S1 Table Custom-designed DNA Microarray Gene List. (XLSX)
