# Supplementary figures and images for: Nucleosome Repositioning: A Novel Mechanism for Nicotine- and Cocaine-Induced Epigenetic Changes
Source: PLoS One. 2015 Sep 28;10(9):e0139103. doi: 10.1371/journal.pone.0139103 (PMC4586372; doi:10.1371/journal.pone.0139103)

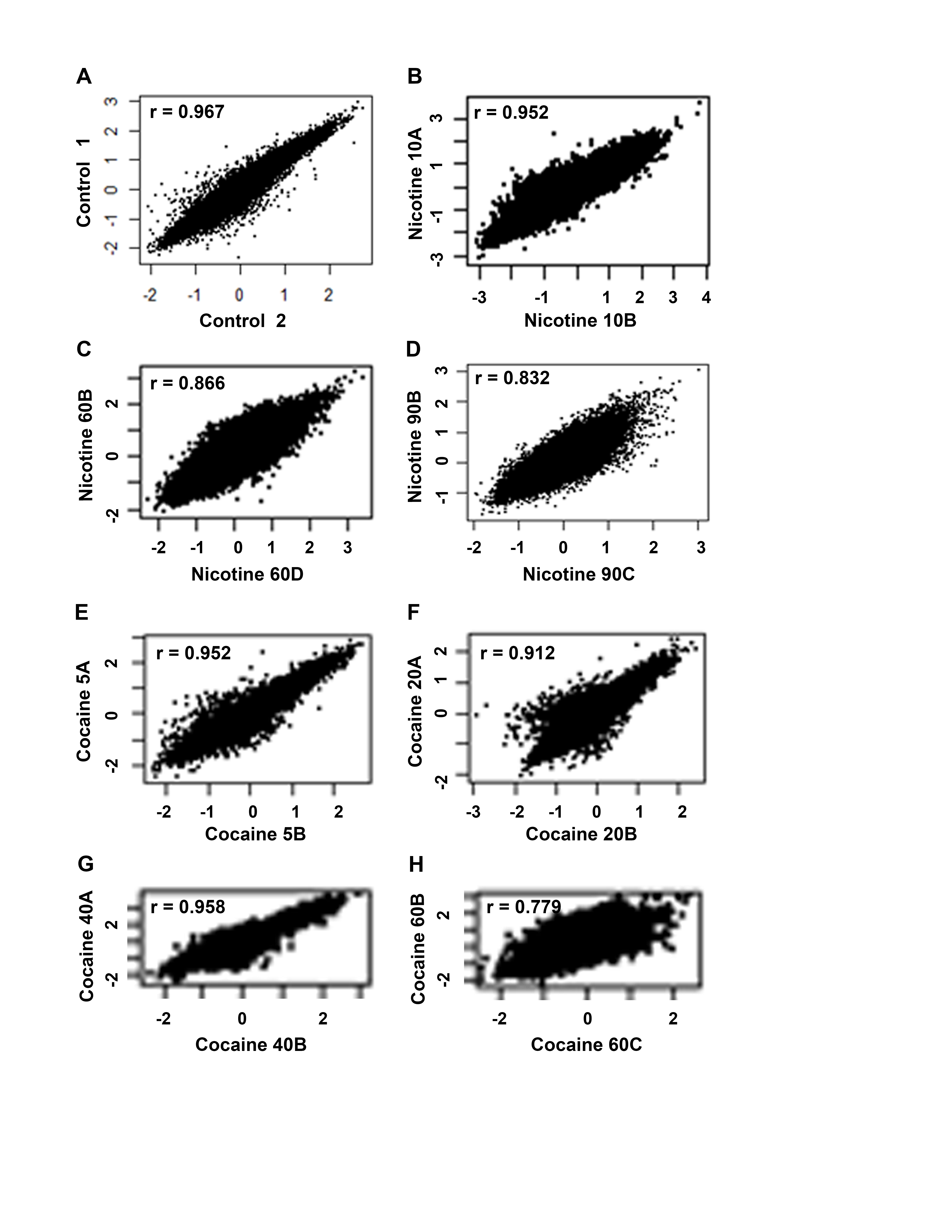

Supplement: S1 Fig — (A–H) Scatterplots for biological replicates for each drug and every time point demonstrate the correlation between replicates shown by the corresponding r-values. The x- and y-axis represent the log2 ratio of nucleosomally-protected DNA to genomic DNA signal at each probe on the microarray. (TIF) [file pone.0139103.s001.tif]

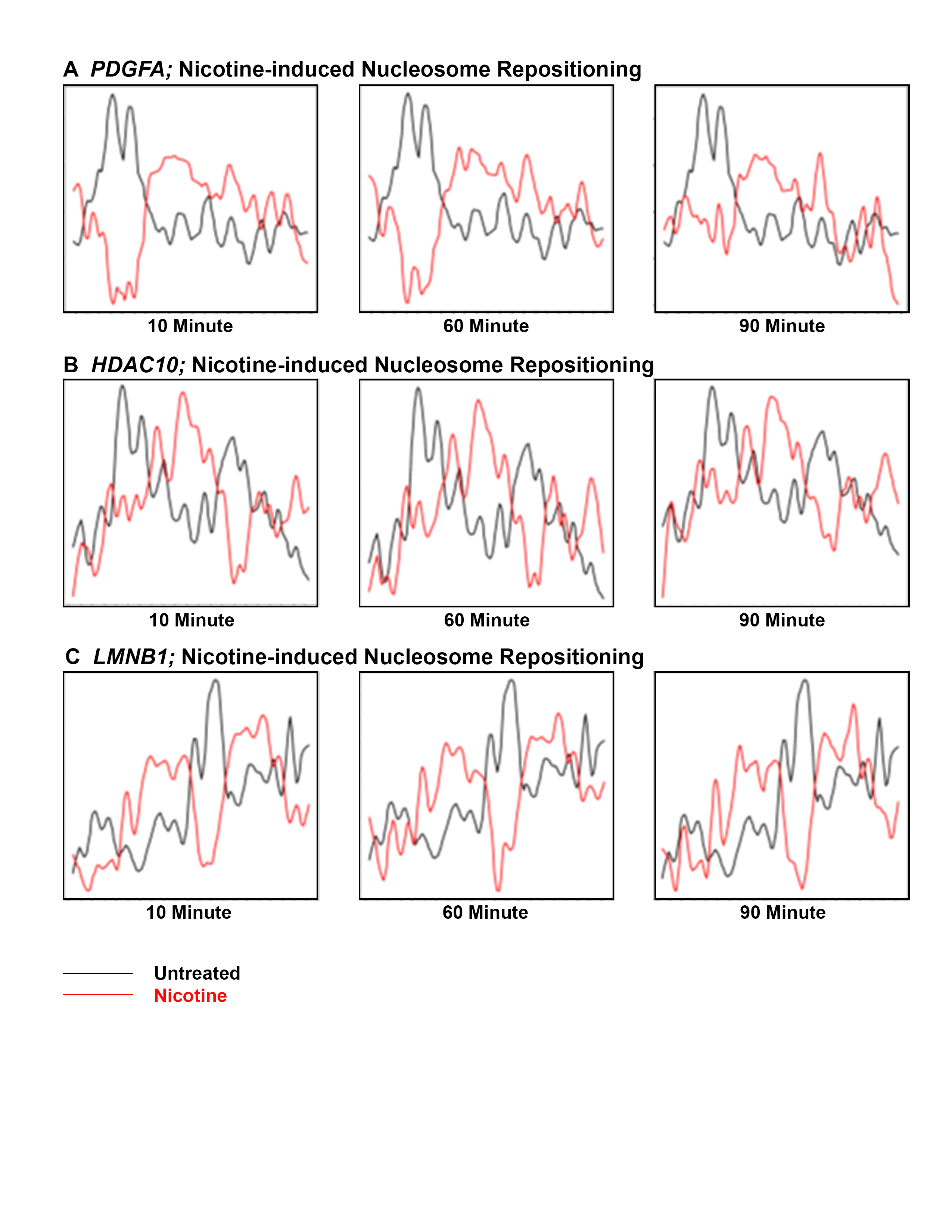

Supplement: S2 Fig — Persistent repositioning of nucleosomes is seen for (A) PDGFA, (B) HDAC10 and (C) LMNB1 genes following 10, 60 and 90 min of nicotine exposure. Changes in nucleosome position are depicted as the divergence of the red and black lines. The black line is the basal nucleosome occupancy signal in the drug naïve state. The red line is the nucleosome occupancy signal following nicotine exposure. Changes in nucleosome occupancy for all three genes were observed at 10 min and persisted through the 60 and 90 min time points with few modifications. (TIF) [file pone.0139103.s002.tif]
